# Irisin activates Opa1-induced mitophagy to protect cardiomyocytes against apoptosis following myocardial infarction

**DOI:** 10.18632/aging.102899

**Published:** 2020-03-10

**Authors:** Ting Xin, Chengzhi Lu

**Affiliations:** 1 The First Center Clinic College of Tianjin Medical University, Tianjin First Center Hospital, Tianjin, China; 2 Department of Cardiology, Tianjin First Center Hospital, Tianjin, China

**Keywords:** Opa1, irisin, myocardial infarction, mitophagy, mitochondria

## Abstract

Myocardial infarction is characterized by sudden ischemia and cardiomyocyte death. Mitochondria have critical roles in regulating cardiomyocyte viability and can sustain damage under ischemic conditions. Mitophagy is a mechanism by which damaged mitochondria are removed by autophagy to maintain mitochondrial structure and function. We investigated the role of the dynamin-like GTPase optic atrophy 1 (Opa1) in mitophagy following myocardial infarction. Opa1 expression was downregulated in infarcted hearts *in vivo* and in hypoxia-treated cardiomyocytes *in vitro*. We found that Opa1 overexpression protected cardiomyocytes against hypoxia-induced damage and enhanced cell viability by inducing mitophagy. Opa1-induced mitophagy was activated by treatment with irisin, which protected cardiomyocytes from further damage following myocardial infarction. Opa1 knockdown abolished the cardioprotective effects of irisin resulting in an enhanced inflammatory response, increased oxidative stress, and mitochondrial dysfunction in cardiomyocytes. Our data indicate that Opa1 plays an important role in maintaining cardiomyocyte viability and mitochondrial function following myocardial infarction by inducing mitophagy. Irisin can activate Opa1-induced mitophagy and protect against cardiomyocyte injury following myocardial infarction.

## INTRODUCTION

Myocardial infarction is characterized by sudden ischemia, cardiomyocyte death, and fibrosis [1, 2]. Many signaling pathways can cause cardiomyocyte death including the mitochondrial apoptosis pathway and the inflammatory response [3]. Antiapoptotic and antioxidative agents have been shown to reduce cardiomyocyte damage [4]. Additionally, therapeutic strategies that target the mitochondria in damaged cardiomyocytes may protect against cardiomyocyte death following myocardial infarction [5, 6]. Damaged mitochondria release reactive oxygen species (ROS) into the cytoplasm, which can lead to apoptosis [7]. Mitochondria are important for producing ATP to sustain cardiomyocyte contractility and function. They also release pro-apoptotic factors to initiate programmatic cell death and are therefore critical regulators of cell face and function [8, 9].

Mitophagy is a mechanism by which damaged mitochondria are removed by autophagy to maintain mitochondrial structure and function. Mitophagy can block the release of ROS release from mitochondria and prevent apoptosis following mitochondrial damage in a lysosome-dependent process [10, 11]. Optic atrophy 1 (Opa1) is a dynamin-like GTPase that regulates fusion of the mitochondrial inner membrane [12]. Opa1 has been shown to regulate mitophagy through fusion-dependent and -independent mechanisms [13]. Interestingly, Opa1 has been shown to have cardioprotective effects [14]. Increased Opa1 expression was shown to reduce oxidative stress in cardiomyocytes in hypoxia-reperfusion injury through Ca^2+^/calmodulin-dependent protein kinase II (CaMKII signaling) [15]. Additionally, Opa1 upregulation reduced myocardial ischemia through activation of the Brain-derived neurotrophic factor (BDNF)/tropomyosin-related kinase B (TrkB) pathway [16]. Reduced Opa1 expression inhibited mitophagy resulting in myocardial ischemia-reperfusion injury [17]. Finally, inhibition of mitophagy through PTEN-dependent upregulation of Opa1 protected cardiomyocytes against lipopolysaccharide (LPS)-mediated inflammation in a model of septic cardiomyopathy [18–20]. Thus, Opa1 may play a role in protecting cardiomyocytes from damage following myocardial infarction by regulating mitophagy [21, 22].

Mitophagy is important for regulating mitochondrial energy metabolism, oxidative stress, and apoptosis [23, 24]. Here, we investigated whether Opa1 promotes mitophagy to protect cardiomyocytes following myocardial infarction. Additionally, we explored whether irisin, a drug that has been shown to regulate mitochondrial function and suppress septic cardiomyopathy, could regulate Opa1-induced mitophagy following myocardial infarction [25–28].

## RESULTS

### Opa1 is downregulated in the infarcted heart and in hypoxia-treated cardiomyocytes

**Figure 1:**
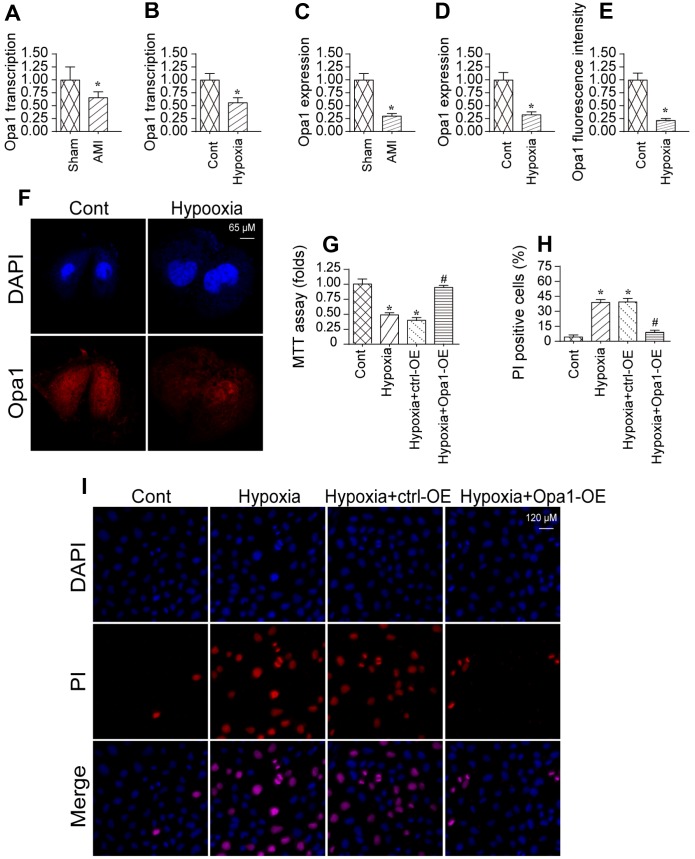
**Opa1 is downregulated in the infarcted heart and in hypoxia-treated cardiomyocytes.** (**A**–**D**) Quantitative real-time PCR and western blot analysis of Opa1 expression. (**E**, **F**) Analysis of Opa1 expression in cardiomyocytes *in vitro* by immunofluorescence. (**G**) MTT assays of cardiomyocyte viability. (**H**, **I**) Analysis of cardiomyocyte apoptosis by PI staining. *P < 0.05 vs. the control group; #P < 0.05 vs. the hypoxia + ctrl-OE group.

We previously established an *in vivo* model of myocardial infarction [29, 30]. Using that model, we analyzed Opa1 expression in infarcted hearts compared to controls using quantitative real-time PCR and western blotting. Opa1 expression was lower in infarcted hearts compared to controls (sham) indicating Opa1 expression is downregulated following myocardial infarction (Figure 1A–1D). A reduction in Opa1 expression was also observed by immunofluorescence.

We also established an *in vitro* model of myocardial infarction in which cardiomyocytes were subjected to hypoxic conditions. Opa1 expression was reduced in cardiomyocytes cultured under hypoxic conditions for 48 hours compared to controls (Figure 1E, 1F), which is consistent with those of a previous findings [31]. Overexpression of Opa1 increased the viability hypoxia-treated cardiomyocytes as compared to untransfected controls (Figure 1G). Correspondingly, Opa1 overexpression in reduced the incidence of apoptosis among hypoxia-treated cardiomyocytes (Figure 1H–1I). These data suggest that Opa1 is important for protecting cardiomyocytes against hypoxia-induced damage.

### Opa1 mediates mitophagy in the infarcted heart

**Figure 2:**
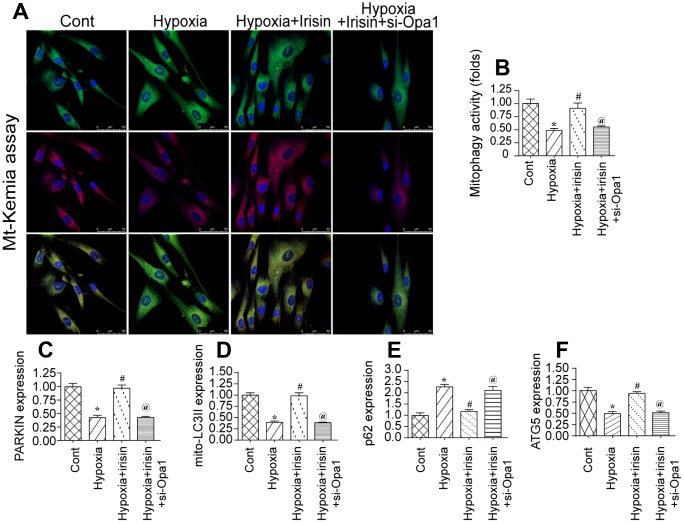
**Irisin activates Opa1-induced mitophagy.** (**A**, **B**) Flow cytometry analysis of mitophagy using the fluorescent probe mt-Keima. (**C**–**F**) Analysis of the expression of mitophagy-associated proteins by western blotting. *P < 0.05 vs. the control group; #P < 0.05 vs. the hypoxia + irisin group.

Previous studies demonstrated that Opa1 can promote mitophagy [32, 33]. We therefore investigated the effect of hypoxia on mitophagy in cardiomyocytes by flow cytometry using the fluorescent reporter mt-Keima. We observed a reduction in mitophagy in hypoxia-treated cardiomyocytes compared to controls (Figure 2A, 2B). Interestingly, Opa1 overexpression resulted in an increase in mitophagy in hypoxia-treated cardiomyocytes, suggesting it may induce mitophagy in the infarcted heart [34, 35].

We previously demonstrated that irisin modulated mitochondrial function in a model of septic cardiomyopathy. We therefore hypothesized that irisin could modulate Opa1-induced mitophagy in hypoxia-treated cardiomyocytes following myocardial infarction. Interestingly, we observed a decrease in the levels of various mitophagy-associated proteins under hypoxic conditions by western blotting. This effect was reversed by treatment with irisin, suggesting that irisin can activate Opa1-induced mitophagy in cardiomyocytes under hypoxic stress (Figure 2C–2F).

### Irisin activates Opa1-induced mitophagy and restores mitochondrial energy metabolism

**Figure 3:**
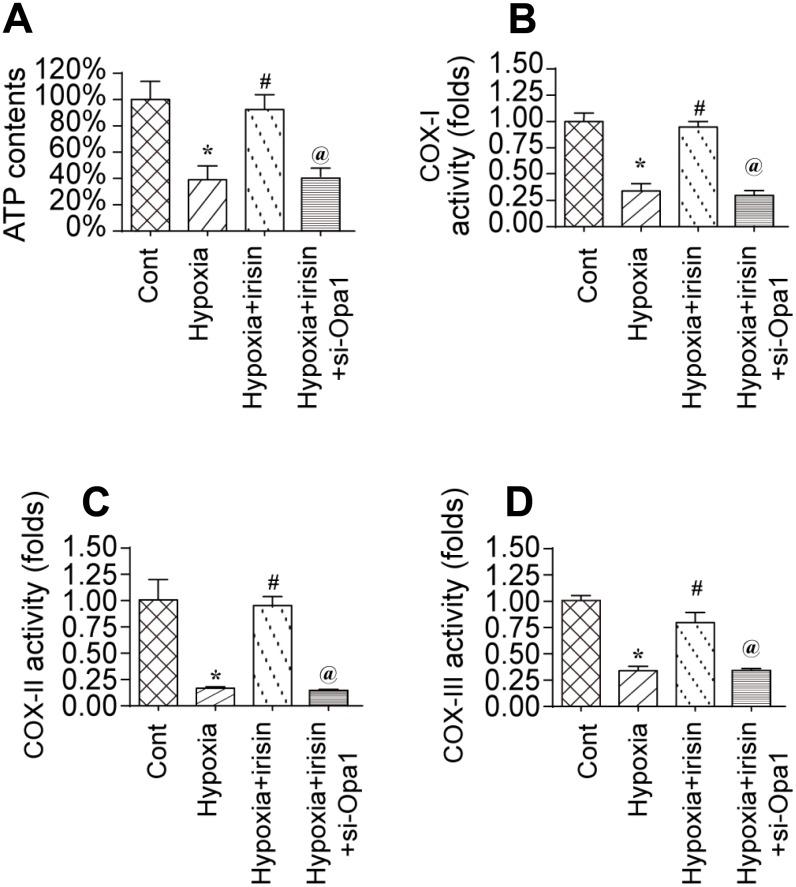
**Irisin activates Opa1-induced mitophagy to restore mitochondrial energy metabolism.** (**A**) Measurement of ATP production by ELISA. (**B**–**D**) Measurement of mitochondrial respiratory complex activity by ELISA. *P < 0.05 vs. the control group; #P < 0.05 vs. the hypoxia + irisin group.

To investigate the mechanisms underlying the protective effects of Opa1-induced mitophagy, we evaluated the alterations in mitochondrial function [36]. A reduction in the levels of mitochondria-derived ATP was observed in hypoxia-treated cardiomyocytes. Treatment with irisin resulted in an increase in ATP levels in hypoxia-treated cardiomyocytes compared to controls (Figure 3A) [37, 38]. The increase in ATP was inhibited by knockdown of Opa1 by siRNA (si-Opa1) (Figure 3A). We also observed downregulation of the levels of the mitochondrial respiratory complex in response to hypoxia, which was reversed by treatment with irisin (Figure 3B–3D). Knockdown of Opa1 by siRNA abolished the irisin-mediated protective effects on the mitochondrial respiratory complex (Figure 3B–3D). These results indicate that irisin exerts cardioprotective effects by activating Opa1-induced mitophagy.

### Opa1-induced mitophagy maintains mitochondrial function and reduces oxidative stress

**Figure 4:**
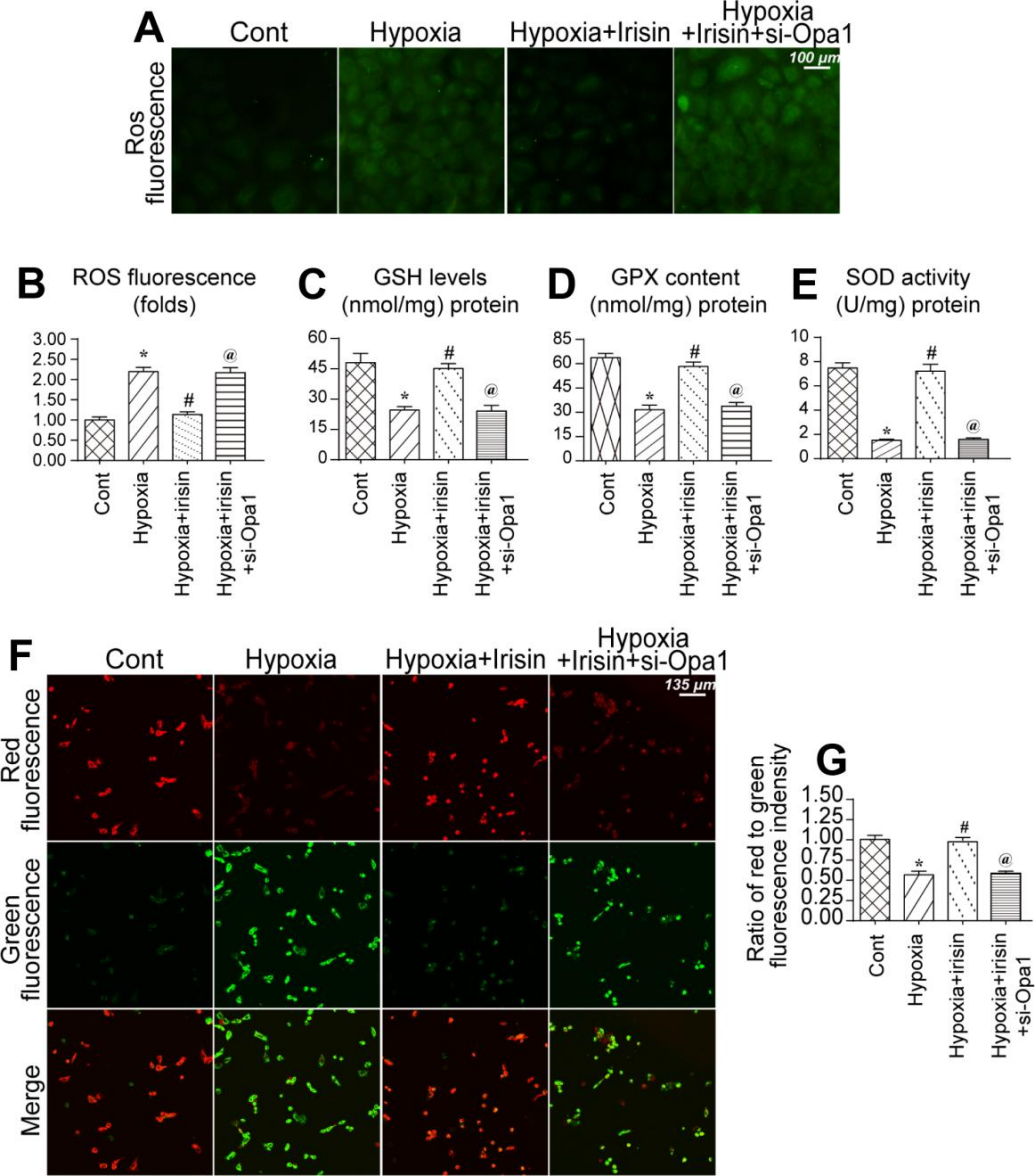
**Opa1-induced mitophagy maintains mitochondrial function and reduces oxidative stress.** (**A**, **B**) Analysis of ROS levels in cardiomyocytes. (**C**–**E**) ELISA assays to evaluate the levels of antioxidants. (**F**, **G**) Measurement of alterations in the mitochondrial membrane potential using a JC-1 probe. *P < 0.05 vs. the control group; #P < 0.05 vs. the hypoxia + irisin group.

We further analyzed the protective effects of irisin and Opa1-induced mitophagy following myocardial infarction [39, 40]. An increase in ROS in mitochondria was observed in hypoxia-treated cardiomyocytes (Figure 4A, 4B). Irisin reduced the levels of ROS whereas Opa1 knockdown by siRNA suppressed the antioxidative effects of irisin in hypoxia-treated cardiomyocytes (Figure 4A, 4B). Additionally, we found that the levels of components of the antioxidative system including glutathione (GSH), superoxide dismutase (SOD), and glutathione peroxidase (GPX), were reduced under conditions of hypoxic stress (Figure 4C–4E). Interestingly, irisin promoted Opa1-induced mitophagy and increased the levels of GSH, SOD, and GPX (Figure 4C–4E). Thus, irisin promotes Opa1-induced mitophagy, which reduces oxidative stress.

We also found that the mitochondrial membrane potential, a marker of mitochondrial function, was disrupted by hypoxic stress (Figure 4F, 4G) [41, 42]. Irisin treatment restored the mitochondrial membrane potential in hypoxia-treated cardiomyocytes whereas Opa1 knockdown disrupted the mitochondrial membrane potential in irisin-treated cardiomyocytes (Figure 4F, 4G). These data indicate Opa1-induced mitophagy is important for maintaining mitochondrial function and reducing oxidative stress in cardiomyocytes.

### Opa1-induced mitophagy inhibits apoptosis

**Figure 5:**
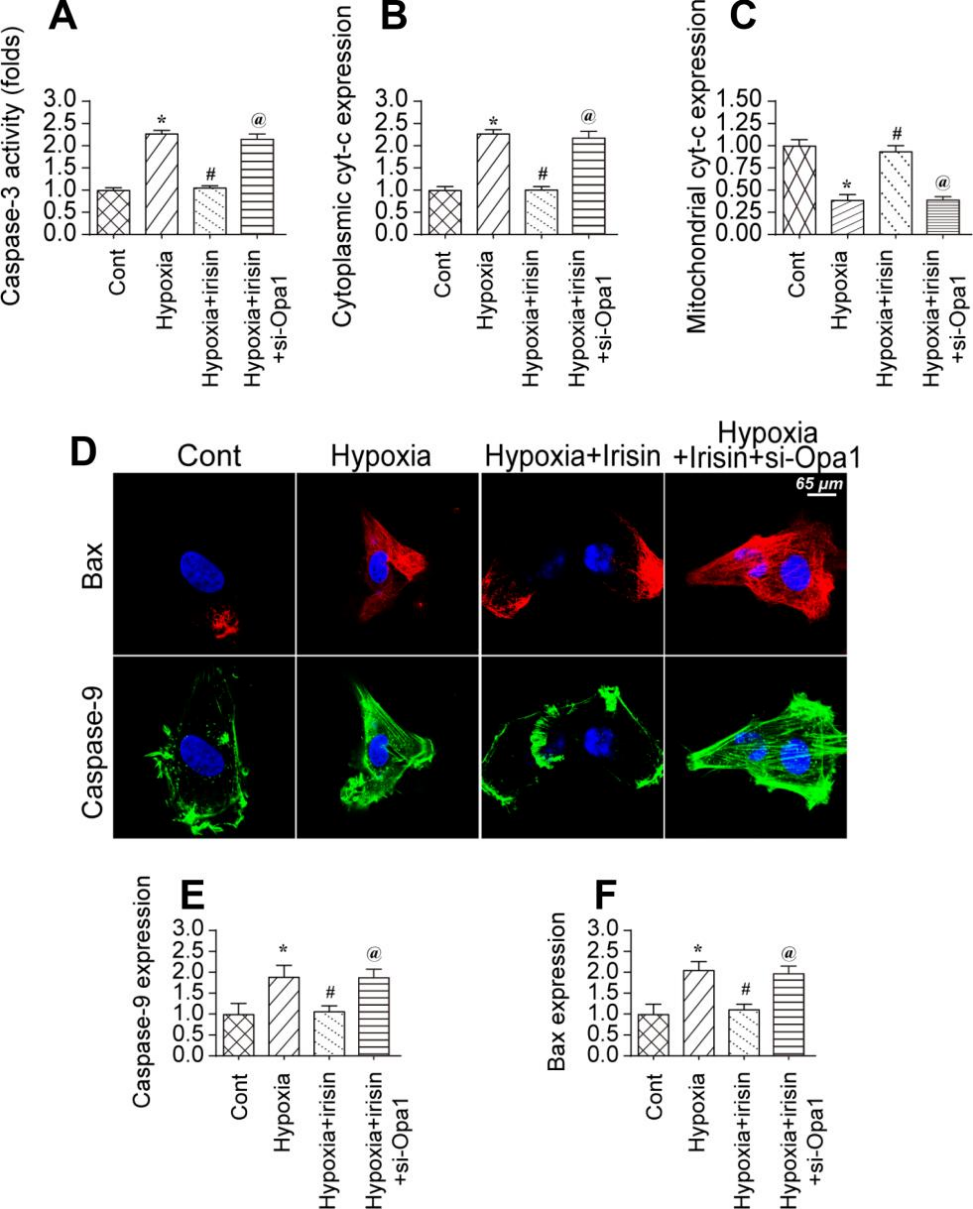
**Opa1-induced mitophagy inhibits the mitochondrial apoptosis pathway.** (**A**) Analysis of caspase-3 activity in cardiomyocytes by ELISA. (**B**, **C**) Western blot analysis of cyt-c levels in the cytoplasm and mitochondria. (**D**–**F**) Immunofluorescence analysis of Bax and caspase-9 expression in cardiomyocytes. *P < 0.05 vs. the control group; #P < 0.05 vs. the hypoxia + irisin group.

We investigated whether Opa1-induced mitophagy could protect cardiomyocytes against hypoxia-induced apoptosis [43]. Caspase-3 activity increased in hypoxia-treated cardiomyocytes, which was indicative of activation of apoptotic cell death (Figure 5A) [44]. Treatment of hypoxia-treated cardiomyocytes with irisin inhibited caspase-3 activation. Finally, knockdown of Opa1 enhanced caspase-3 activation in irisin-treated cardiomyocytes (Figure 5A). These data indicate that the antiapoptotic effects of irisin are mediated by Opa1-induced mitophagy.

The mitochondrial apoptotic pathway is characterized by cytochrome C (cyt-c) translocation to the cytoplasm. We observed an increase in cytoplasmic cyt-c levels by western blotting in hypoxia-treated cardiomyocytes (Figure 5B, 5C). Irisin treatment resulted in a decrease in the levels of cytoplasmic cyt-c in an Opa1-dependent manner (Figure 5B, 5C). The mitochondrial apoptotic pathway is also characterized by upregulation of caspase-9 and Bax [45]. We observed upregulation of both caspase-9 and Bax in hypoxia-treated cardiomyocytes by immunofluorescence (Figure 5D–5F). Treatment with irisin resulted in downregulation of caspase-9 and Bax in hypoxia-treated cardiomyocytes (Figure 5D–5F). Knockdown of Opa1 by siRNA resulted in an increase in caspase-9 and Bax levels in irisin- and hypoxia-treated cardiomyocytes (Figure 5D–5F). These data suggest that the anti-apoptotic effects of irisin require activation of Opa1-induced mitophagy in hypoxia-treated cardiomyocytes.

## DISCUSSION

Cardiomyocyte damage and death contributes to the pathogenesis of myocardial infarction [46, 47]. Myocardial infarction is caused by the sudden loss of blood supply to the heart and leads to cardiomyocyte death [48–50]. Death of cardiomyocytes results in activation of the inflammatory response [51, 52]. Additionally, the lack of blood flow and nutrients activates the transcription of pro-inflammatory factors that further enhance inflammation of the myocardium [53, 54]. Excessive inflammation can lead to the accumulation of pro-inflammatory cells such as white blood cells [55]. Although white blood cells are involved in repairing damage to the myocardium, they can also induce oxidative stress [56, 57]. The accumulation of these pro-inflammatory factors increases cardiomyocyte damage [58, 59].

Both mitochondria-dependent and -independent apoptotic pathways can lead to cardiomyocyte death [60, 61]. The mitochondria-dependent pathway is initiated in response to unrepaired mitochondrial damage. In contrast, the mitochondria-independent pathway is regulated by the endoplasmic reticulum [62, 63], lysosomes [64], and other factors. There are several differences between the mitochondria-dependent and -independent apoptosis pathways [65, 66]. The mitochondria-dependent pathway involves a reduction in mitochondrial membrane potential and activation of caspase-9 whereas the mitochondria-independent pathway is characterized by activation of caspase-3 and cellular membrane rupture [67]. We found that mitochondria-dependent apoptosis occurs during the progression of myocardial infarction. However, we could not exclude the possibility of mitochondria-independent apoptosis [68, 69]. We also found that the inflammatory response and oxidative stress were activated following myocardial infarction and could trigger cardiomyocyte death through the mitochondria-dependent apoptosis pathway [70].

Several studies have explored approaches for blocking mitochondria-dependent apoptosis in cardiomyocytes [71]. Mitophagy is a protective mechanism that involves the selective removal of dysfunctional mitochondria thereby allowing cellular repair [72]. Several proteins that are important for autophagy also play a role in mitophagy [73]. However, there are some proteins that are only involved in mitophagy including FUNDC1, BNIP3, PARK2, and NIX [74]. These proteins may have roles in cardiovascular diseases including myocardial infarction.

We previously demonstrated that the inner mitochondrial membrane protein Opa1 has an important role in promoting mitophagy [75, 76]. Consistent with previous studies, we found that irisin could activate Opa1-induced mitophagy in cardiomyocytes [77, 78]. Irisin treatment was associated with an increase in Opa1 expression. However, we did not evaluate whether there are differences in the expression of the Opa1 isoforms (L-Opa1 and S-Opa1) [79, 80]. Interestingly, Opa1 knockdown suppressed mitophagy and abolished the cardioprotective effects of irisin, suggesting that the protective effects of irisin on cardiomyocytes following myocardial infarction are dependent upon Opa1-induced mitophagy [81, 82].

Collectively, our data indicate that Opa1 plays an important role in regulating cardiomyocyte viability following myocardial infarction by activating mitophagy. Irisin can activate Opa1-induced mitophagy in the infarcted heart and could have therapeutic efficacy in patients with acute myocardial injury.

## MATERIALS AND METHODS

### Cell culture and animal models

Primary cardiomyocytes were isolated from and a model of myocardial infarction established as described [83, 84]. Cells were cultured under hypoxic conditions for 48 hours to mimic myocardial infarction [24, 85]. Cardiomyocytes were cultured in Dulbecco’s Modified Eagle Medium (DMEM, Sigma, St. Louis, MO, USA) supplemented with 10% fetal bovine serum (Sigma, St. Louis, MO, USA) in a humidified atmosphere of 5% CO_2_ at 37°C. Irisin treatment was performed as described previously [86, 87].

### Immunofluorescence staining

Cells seeded in plates were fixed with 4% paraformaldehyde and then blocked and permeabilized in solution containing 3% BSA (Sigma Aldrich, St. Louis, MO, USA), 10% normal goat serum (Vector Laboratories, Burlingame, CA, USA), and 0.3% Triton X-100 (Sigma Aldrich, St. Louis, MO, USA). The cells were then incubated with the indicated primary antibodies (Cell Signaling Technology, Danvers, MA, USA; Abcam, Cambridge, MA, USA) [88, 89]. Following the incubation, the cells were washed and incubated with corresponding Alexa Fluor secondary antibodies (Life Technologies, Carlsbad, CA, USA) [90, 91]. Lipid droplets and nuclei were stained with Hoechst 33342 prior to imaging the cells by confocal microscopy [92].

### ROS measurement

Intracellular ROS levels were measured in cells plated in 6-well dishes at an equal density. The cells were trypsinized, washed with phosphate-buffered saline (PBS) [93, 94], and then stained with 2 μmol/L chloromethyl-20,70-dichlorodihydrofluorescein diacetate (CM-H_2_-DCFDA) (Life Technologies, Carlsbad, CA, USA). Relative cell counts were determined using a FACSCanto II Analyzer flow cytometer (BD Biosciences, San Jose, CA, USA). Data were analyzed using the FlowJo software (FlowJo, LLC, Ashland, OR, USA) [95].

### Opa1 knockdown and overexpression

Opa1 knockdown was performed using siRNA as described [96–99]. Briefly, cardiomyocytes were cultured in 6-well plates (1 x 10^6^ / well), 6 cm dishes (3 x 10^6^ / dish), or petri dishes (3 x 10^5^ / well) [100]. The siRNA dose was the following: 50 pmol / petri dish, 100 pmol / 6-well plates, 166 pmol / 6 cm dish, and 434 pmol / 10 cm dish. Cell transfection with siRNA was performed using the Lipofectamine RNAiMAX Reagent (Invitrogen, Carlsbad, CA, USA) according to the manufacturer’s instructions [101]. Knockdown efficiency was evaluated 36 hours after transfection by quantitative real-time PCR. Adenovirus overexpression assays were performed as described previously [102].

### Western blotting

Cells were washed with PBS and lysed in cold lysis buffer containing a protease inhibitor cocktail as described [103, 104]. Cell lysates were centrifuged at 13,000 g at 4 °C for 40 min. The supernatants were collected and total protein quantified [105]. Equal quantities of total protein were separated by 10% SDS-PAGE and transferred to nitrocellulose membranes (Millipore, Bedford, MA, USA). The membranes were incubated with primary antibodies at 4°C for 15 hours [106]. Following the incubation, the membranes were incubated with the respective secondary antibodies at 25°C for 90 minutes. Proteins were visualized using the ECL substrate (Thermo Fisher Scientific, Waltham, MA, USA). GAPDH was used as a loading control [107, 108].

### Quantitative real-time PCR

RNA from serum was extracted using the TRIzol reagent (Invitrogen, Carlsbad, CA, USA) according to the manufacturer’s protocol [109, 110]. The mRNA was reverse transcribed into cDNA using the First Strand Synthesis Kit (Thermo Fisher Scientific, Waltham, MA, USA) [111]. Real-time PCR was performed using an ABI7900 Real-time PCR system (Applied Biosystems, Foster City, CA, USA) using the SYBR Green Master Mix Kit (Takara, Dalian, China) [112]. We normalize mRNA expression to that of GAPDH. Relative gene expression was calculated using the comparative Ct method [113].

### Enzyme-linked immunosorbent assays

The levels of antioxidants such as GSH, SOD, and GPX were evaluated using enzyme-linked immunosorbent assay (ELISA) kits (Abcam, Cambridge, MA, USA) according to the manufacturer’s instructions [112, 114].

### Statistical analysis

Quantitative real-time PCR and ELISAs data were analyzed using GraphPad Prism 5 (GraphPad Software, La Jolla, CA, USA) [115]. The data are presented as the mean ± standard deviation. Differences between groups were analyzed using unpaired t-tests. *A P < 0.05 was considered statistically significant [116].
